# IoT platforms assessment methodology for COVID-19 vaccine logistics and transportation: a multi-methods decision making model

**DOI:** 10.1038/s41598-023-44966-y

**Published:** 2023-10-16

**Authors:** Yasir Ali, Habib Ullah Khan

**Affiliations:** 1 Shahzeb Shaheed Government Degree College Razzar, Swabi, Higher Education Department, Peshawar, Khyber Pakhtunkhwa Pakistan; 2 Accounting and Information Systems, College of Business and Economics, Qatar University, Doha, Qatar

**Keywords:** Computer science, Disease prevention

## Abstract

The supply chain management (SCM) of COVID-19 vaccine is the most daunting task for logistics and supply managers due to temperature sensitivity and complex logistics process. Therefore, several technologies have been applied but the complexity of COVID-19 vaccine makes the Internet of Things (IoT) a strong use case due to its multiple features support like excursion notification, data sharing, connectivity management, secure shipping, real-time tracking and monitoring etc. All these features can only feasible through choosing and deploying the right IoT platform. However, selection of right IoT platform is also a major concern due to lack of experience and technical knowledge of supply chain managers and diversified landscape of IoT platforms. Therefore, we introduce a decision making model for evaluation and decision making of IoT platforms that fits for logistics and transportation (L&T) process of COVID-19 vaccine. This study initially identifies the major challenges addressed during the SCM of COVID-19 vaccine and then provides reasonable solution by presenting the assessment model for selection of rational IoT platform. The proposed model applies hybrid Multi Criteria Decision Making (MCDM) approach for evaluation. It also adopts Estimation-Talk-Estimation (ETE) approach for response collection during the survey. As, this is first kind of model so the proposed model is validated and tested by conducting a survey with experts. The results of the proposed decision making model are also verified by Simple Additive Weighting (SAW) technique which indicates higher results accuracy and reliability of the proposed model. Similarly, the proposed model yields the best possible results and it can be judged by the precision, accuracy and recall values i.e. 93%, 93% and 94% respectively. The survey-based testing also suggests that this model can be adopted in practical scenarios to deal with complexities which may arise during the decision making of IoT platform for COVID-19 SCM process.

## Introduction

Infectious diseases such as chickenpox, measles and pneumonia can only be controlled by vaccination^1^. COVID-19 disease can only be handled by proper vaccination drive. In this regard, majority of countries started the administration of the vaccine to uphold the health condition and immunization of their citizens. However, the process of vaccine administration is hampered by demographical factor, less technological development and lack of proper vaccine cold chain management especially in third world countries. These challenges have emerged immense issues to cover the largest population quickly and in a timely fashion. Similarly, vaccines are not similar to other drugs as they require proper monitoring and tracking. In this regard, World Health Organization (WHO) released guidelines to develop, monitor, receive, store, manage, distribute and strengthen the supply chain process. The COVID-19 vaccine require accuracy, safety, efficacy, proper utilization and tracking through the entire process of supply chain operations. But, in the current pandemic, during the vaccine roll out many challenges have been faced by healthcare professionals and pharmaceutical industries that require fast and reliable solutions, otherwise the battle of COVID-19 will be hard to win. COVID-19 pandemic has affected the business, lives and economy all over the world^2^. It has brought sever disruption in supply chain but its impact in healthcare supply chain is significant^3^. The current COVID-19 pandemic also triggered the need of internet connectivity and e-platforms due to network congestion^4^. The supply chain managers require persistent connection to carry on the online purchasing and daily operations. This pandemic has not only affected road transportation by it also have disrupted the air transportation as well^5,6^.

The L&T of the COVID-19 vaccine is a complex process because of the number of factors involved such as the number of entities, ultra-cooling freezing, testing of delivery procedure, nature of material used and organisations. These factors lead to bottlenecks and challenges such as integrity, traceability, logistic/cold chain management, lack of personnel, lack of real-time information or misinformation about tracking, illegal diversion/theft, vaccine tempering, lack of coordination, lack of supplies and manufacturing delays. COVID-19 outbreak has brought many challenges for logistics companies^7^. Among the challenges, the integrity issue is important because it describes how the vaccine is transported from the manufacturing place to the point of administration while maintaining its original shape. For example, some vaccines require a threshold value of temperature and should not exceed this value. All vaccines are sensitive to heat and must be stored at one degree Fahrenheit or below the temperature. For example, some types of vaccines like mRNA can lose efficiency at variable temperatures and are requiring a cold chain of storage and transportation. Furthermore, the SCM of COVID-19 vaccine is different from the traditional vaccine, so it has put healthcare professionals and supply chain managers into a dilemma to provide better strategies and policies to combat COVID-19^8^. These challenges are not allowing the healthcare professionals and supply chain managers to cover the target population and also to reach out the maximum people in short time. The conventional approach towards the development, L&T and administration of vaccine will not be the right solution. Therefore, it is imperative to have a digital solution to provide a better and fast supply chain of COVID-19 vaccine in less time to the maximum number of people.

The L&T of COVID-19 vaccine is a tedious task due to many entities involved. This process is also hampered by many challenging factors like quick delivery, cold storage management, improper routing, lack of planning and many others. During the COVID-19 pandemic, the vaccine transportation and shipping created many challenges for the fleet and shipping managers. However, addressing these challenges manually can be tedious and herculean task These logistics challenges led to adopt the most befitted technological solution. Different kind of technologies have been applied to expedite the SCM of COVID-19 vaccine during the pandemic and this technological advancement has a significant impact on vaccination drive^9,10^. The leading technologies providing healthcare and supply chain solutions to the challenges posed by the COVID-19 pandemic in the vaccine development, L&T and administration are Robotics, Artificial Intelligence (AI), Edge computing, Blockchain, Computer vision and IoT. Other technologies such as Drone, Big data etc. also have been adopted by shipping companies to leverage the L&T services^11,12^. The application of these modern and smart technologies enhanced the effectiveness and efficiency of L&T systems^13^. Among the contesting technologies, the role of IoT technology is of major importance and noticeable. It has revolutionised almost every sphere of life but its deployment in L&T of COVID-19 vaccine during the pandemic is significant to be highlighted. IoT has shown enormous applications in this area. IoT has enabled the L&T ecosystem by bringing features like secure data sharing, self-driving vehicles and advance automation. IoT has been applied for majority of purposes of shipping products during the pandemic but its use cases increased during the L&T of COVID-19 vaccine. The successful distribution of COVID-19 vaccine from the point of manufacturing to the point of administration requires the best IoT based solution or technology and it is only possible by installing the right IoT platform. This is the reason that IoT technologies have seen enormous growth over the last few years such that many IoT platforms have been introduced in market. IoT platforms can be effective technologies in preserving and monitoring COVID-19 vaccine. IoT platforms provides sensor support where it can be utilized to monitor and track the temperature sensitive vaccine during the SCM cycle^14^. IoT platforms offer the functionalities and services like device management where different sensors can be used for different tasks related to maintaining the safety and potency of COVID-19 vaccine. It can be leveraged to keep eye on the temperature of vaccine during the shipping with the support of Blockchain technology^15^. Blockchain-enabled IoT system can be used to track the vaccine in every phases such as from production line till the administration^16^. The integration of both technologies will allow the SCM stakeholder to track the real time position and quality of vaccine^17^. Similarly, IoT with the collaboration of Artificial Intelligence (AI) can build smart SCM systems for timely delivery, speedy, accurate environment-friendly and justice-based vaccine injection^16^. IoT platforms enable the organizations to perform the shipping and management in less time to meet the organizations objectives^18^.

Keeping all these applications and benefits in view, it is mandatory to pick the most valuable IoT technology that to be deployed in COVID-19 SCM process. All these functionalities and features are only possible due to multi-feature support of IoT platform. The selection of right and apposite platform has major impacts on the sustainability and operationability of shipping companies. IoT platform provides reliable, scalable, optimized performance and secure services to the connected products. IoT platform provides smart logistics and manufacturing by using smart sensors^19^. The rational decision making of choosing right IoT technology among the array of IoT choices is critical for the success of L&T of COVID.19 vaccines. It is also a tough choice to decide about the implementation of IoT technology such that whether it should be adopted in phases, incremented or customized which also has major impacts on the business needs. It is important to analyze the platform vendors by keeping track of history. IoT platforms are complicated as they are interplay of many things like Application Programming Interfaces (APIs), search engines, data analytics and storage, cloud computing services, application development support etc. Choosing IoT platforms haphazardly will bring more problems than the solutions. Therefore, time and resources are required in identifying the sophisticated technology solution for the SCM of COVID-19 vaccine. For this purpose, we introduced a framework using decision making model to evaluate the IoT platforms and pick the right IoT platform to provide smart solution for the COVID-19 vaccine L&T.

### Contribution

The major contributions of the proposed research work are highlighted as.

- This research work identifies the various challenges faced during the manufacturing, cold chain management, logistics and transportation and administration of COVID-19 vaccine. It will help to understand the current research gaps and future directions for research in this area.
- We designed criteria for evaluation based on the important features according to our literature study and surveying experts. This is the toughest part of this research work as we thoroughly searched the entire online databases to collect all features of IoT platform. This procedure is also supported by Delphi method that has been applied for taxonomy and response collection from expert panel in our case study.
- We present IoT platform evaluation framework that is focused on evaluation and decision making related to the ranking and selection of IoT platform or technology for L&T of COVID-19 vaccine. A hybrid decision making approach based on CRiteria Importance Through Intercriteria Correlation (CRITIC) and Technique for Order of Preference by Similarity to Ideal Solution (TOPSIS) techniques have been adopted towards the assessment and decision making of IoT platforms.
- The features and results of the proposed decision making model are tested and verified based on the conducting surveys with two different class of experts and they found it a more suitable and ideal for evaluating and decision making of IoT platforms for the L&T of COVID-19 vaccine. As, this is first kind of evaluation model so the quantitative results were also tested by Simple Additive Weighting (SAW) method.

### Motivation

- The major motivation of presenting the proposed evaluation framework for logistics and transportation of COVID-19 vaccine is that we failed to find any work in well-reputed journals of supply chain management and logistics domain that is focusing on the evaluation of IoT technologies towards the L&T. However, the work presented by Orji et al.^20^ is targeting only on the prioritization of factors (features) related to the deployment of Blockchain technology in the freight logistics industries. They have applied only ANP to rank the features for adoption of Blockchain technology. In comparison to our proposed decision making methodology, the proposed model supports multi-methods and applies hybrid MCDM approach towards the evaluation and selection of IoT platform for L&T of COVID-19 vaccine.
- The L&T companies are facing exorbitant challenges to deliver the COVID-19 vaccine to the target location but the existing technological solutions are not sufficient to provide meaningful solutions. This evaluation framework will provide them a best option to adopt the right IOT based solution for the industrial use and use the optimal features by choosing the best IoT platform.
- We failed to identify any work that is focusing on the IoT platform evaluation and features taxonomy. Similarly, the supply chain managers have less technological background and they are unaware about the IoT platform market and evolving features. Therefore, we were motivated to present this work to provide solutions towards selection issues in IoT platform for shipping of COVID-19 vaccine.

This paper is composed of eight (8) remaining sections: “Related work” Section is related to the literature study. “COVID-19 vaccine and supply chain managment process” Section discusses the COVID-19 vaccine SCM process cycle in detail. “IoT platform evaluation framework for COVID-19 SCM” Section highlights different kinds of issues faced by SCM in its every phase and presents IoT evaluation and decision making model. “Framework evaluation and testing” Section highlights the testing and verification of proposed decision making evaluation model. “Proposed work validation” Section is about the validating the proposed model. “Managerial implications” Section is related to managerial implications of this work. “Study limitations” Section explains the limitations of this research “Conclusion” Section ends with conclusion of this research work.

## Related work

In our literature study, we failed to identify those studies that are focusing on identifying issues related to COVID-19 vaccine transportation and logistics deliver and providing solutions based on using IoT platforms. However, in existing literature different analysis models have been proposed to perform analysis of COVID-19 related data^21–23^. Some evaluation models utilizes statistical approaches to deal with COVID-19 related data^24,25^ while some are AI-based algorithms using Artificial Neural Networks (ANNs) to build simulation model and produces the promising results related to COVID-19 analysis^26^. However, he main focus of this research is to highlight those research studies that are focusing on the analysis and issues related to COVID-19 vaccine during the shipping process. This research collects those studies that are intended to perform the analysis and evaluation of IoT platforms in SCM domain. Literature study of this research work falls into two different categories. In first category, we have identified the different issues and challenges in L&T and in second category, we shed light on decision making approaches related to the selection of IoT platforms in SCM area.

Younan et al.^27^ addressed the challenges and proposed recommended technologies such as ICT in IoT layers in an industrial environment. Ding et al.^28^ highlighted the impact of IoT on supply chain management. This work unfolds the challenges in smart logistics based on IoT and provides research directions for the development of smart logistics. Thibaud et al.^29^ studied IoT-based applications in the environmental, health and safety (EHS) industries. They highlighted challenges in IoT adoption in high-risk EHS industries and proposed solutions to these challenges. The literature review presented by Golpîra^30^ identifies the research gaps and provides guidelines for future research based on the presenting architecture known as Logistics Internet-of-Things (L-IoT). Similarly, Manavalan et al.^31^ presented a conceptual framework based on five management parameters: business, management strategy, sustainable development, technology and collaboration. They explored the prospective opportunities available in IoT-embedded sustainable logistics for Industry 4.0 transformation. Wang et al.^32^ presented IoT-based intelligent logistics system based on cloud and robotics technologies. They applied ant colony and Dijkstra algorithms for supply chain operations.

We also focused on to report the decision making approaches with respect to selection of IoT platform in different sectors to meet the business needs. In this regard, the Kondratenko et al.^33^ evaluated seven (7) IoT platforms by using MCDM approach and among these platforms, they ranked Kaa platform as a best choice for IoT applications. They used eight (8) criteria for decision making purposes such as reliability, device management, level of integration, database functionality, data analytics, protocol support, processing level and action management and visualization usefulness. Similarly, the work presented by M. Ullah et al.^34^ is also based on features and platforms evaluation. They evaluated five IoT platforms such as AWS, Microsoft Azure, IBM Watson, Google cloud an Oracle IoT. They formulated criteria based on twenty-one key features and after the assessment using Delphi method for criteria categorization they concluded that among the selected platform the AWS platform is best of the all. Lin et al.^35^ applied AHP and probabilistic linguistic approach by taking five (5) IoT platforms into account based on features such as scalability, security, usability, market longevity, integration flexibility, pricing and availability. Contreras-Masse et al.^36^ evaluated three IoT platforms based on varying features by using AHP and PROMETHEE-II approach by selecting the Azure IoT as a more suitable and rational choice for IoT solution. Orji et al.^20^ evaluated the factors method that influence the adoption of Blockchian technology by using Analytic Network Process (ANP) method. Mijuskovic et al.^37^ applied AHP method for evaluating five (5) IoT platforms with respect to many functional requirement. Similar works presented by authors for are^38,39^.

## COVID-19 vaccine and supply chain managment process

**Figure 1:**
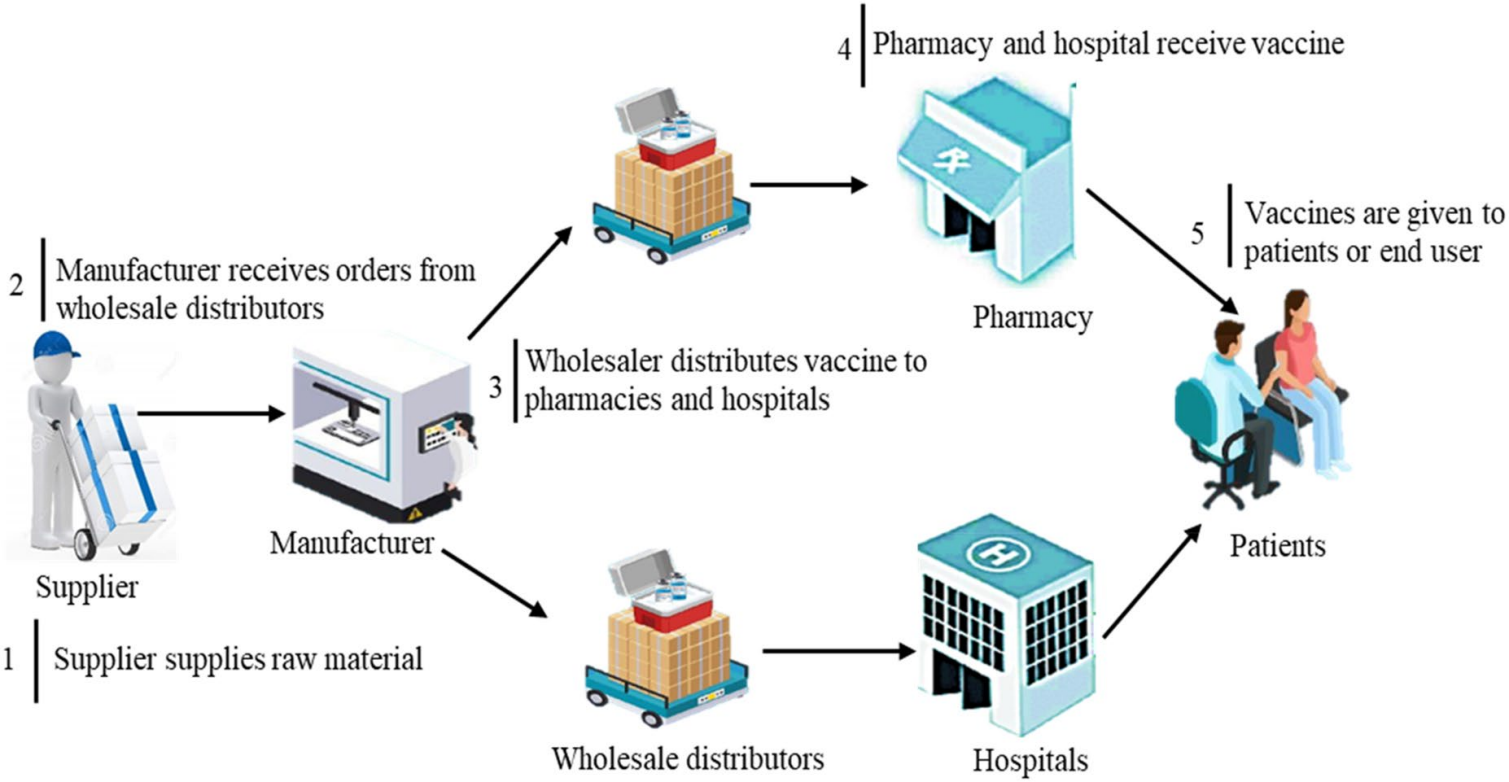
COVID-19 vaccine SCM procedure.

The COVID-19 vaccine SCM process is a complex process as it is not a single activity but composed of a series of different steps/procedures to deliver the vaccine from the manufacturing spot to the final delivery location. A complete picture of the different phases and entities involved in the COVID-19 vaccine SCM model is shown in Fig. 1.

Following are the major actors involved in COVID-19 SCM cycle.

- *Supplier* The COVID-19 supply chain process starts with suppliers, who deliver raw materials to the manufacturers.
- *Manufacturers* They receive orders from wholesalers or distributors, and then the required amount of vaccines is delivered to them after the completion of production.
- *Wholesalers or distributors* These are the people who deliver the product to hospitals or pharmacies
- *Hospitals or pharmacies* These are the stakeholders who buy products from wholesale dealers.
- *Patients* This is the last point, where vaccines are administrated to the patients.

## IoT platform evaluation framework for COVID-19 SCM

**Figure 2:**
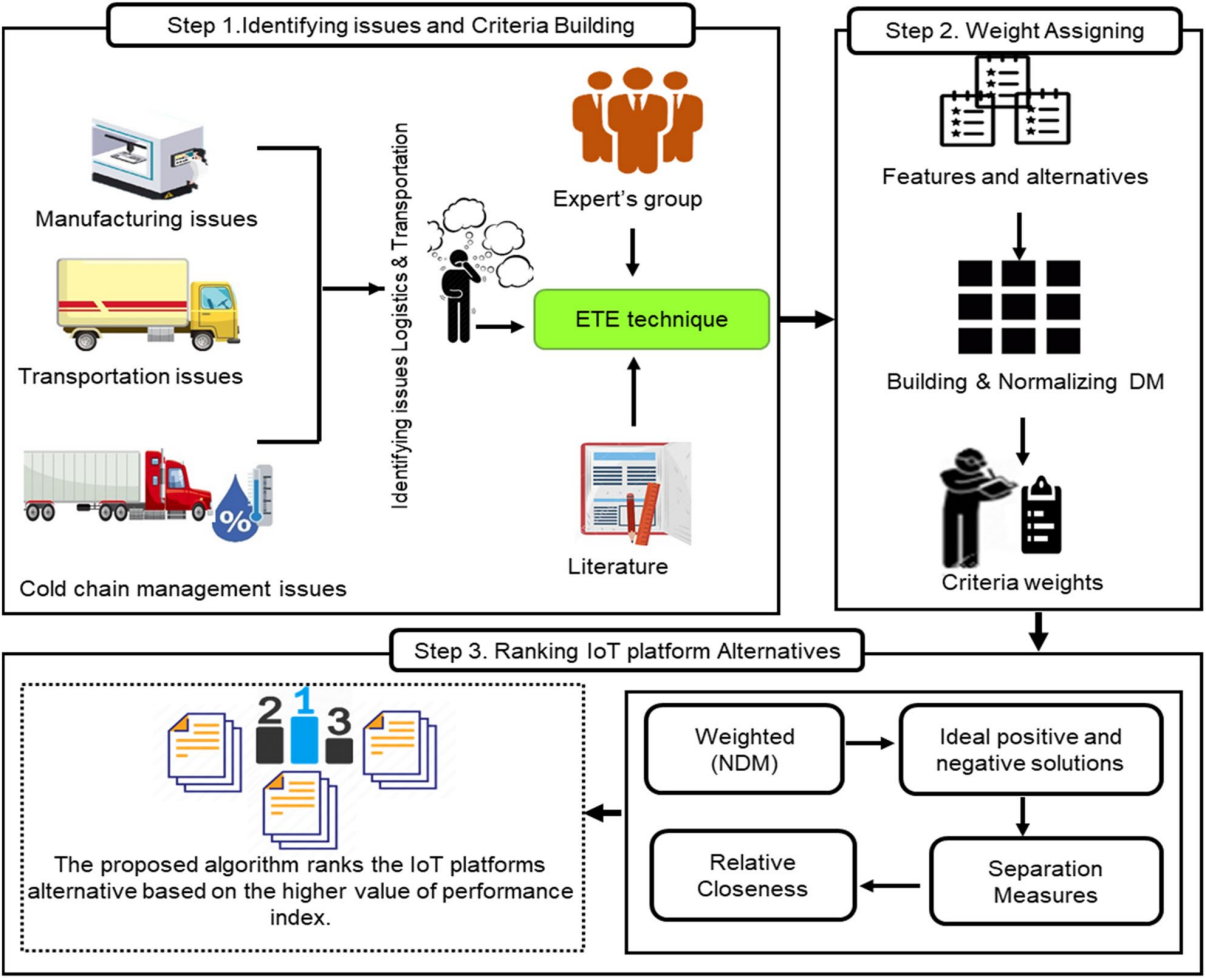
IoT platforms evaluation framework.

The number of IoT platforms has significantly elevated in the recent times. This rapid rise has led to the selection and decision making issue in area of supply chain management. In the current pandemic situation, the selection of right IoT platform that can be used as technological solution of organizational needs is tough task due to bulky list of available platforms employed in L&T system. The selection of right IoT platform also has major impacts on COVID-19 vaccine shipping as it requires technical skills and expertise to choose the right choice among the list of available IoT platforms. The selection and decision making related to IoT platform for supply chain management of COVID-19 vaccine should not be done haphazardly but it should be done based on quantitative data and empirical assessment procedures. To address these selection issues and problems, we propose evaluation framework also known as decision making model for selection of the best IoT platform in L&T of COVID-19 vaccine. The step wise detail of building the proposed evaluation framework is depicted in Fig. 2. Following are the major phases of the proposed evaluation framework of IoT platforms for COVID-19 vaccine L&T.

### Identifying issues in COVID-19 SCM and building criteria

Issues are identified based on literature study and highlighted during the SCM of corona vaccine. The entire SCM process of COVID-19 is hampered by various hiccups and bottlenecks. Different issues need to be addressed for the smooth running of the supply chain process. Unfortunately, these challenges are involved in every phases of SCM such as manufacturing, distribution and transportation, CCM and administration of the COVID-19 vaccine.

#### Manufacturing issues

Vaccine development plays a pivotal role in combating infectious diseases^40^. However, the vaccines are the most complex products as they require hundreds of components for manufacturing. The development of coronavirus vaccines and auxiliary supplies have resulted in a formidable universal challenge. There are a number of pharmaceutical and manufacturing companies that are racing to develop the most effective vaccines. However, vaccine quality should be a major focus of vaccine manufacturing companies. The process of developing vaccine is impeded by many challenges. It is important to identify these challenges and provide tentative solutions to increase the production rate. During the literature study we identified many challenges but the quality is major concern, when it comes to manufacturing of COVID-19 vaccine. The manufacturer of the vaccine must ensure that each vaccine is based on consistent quality and requires repeated testing procedures. Vaccine production is a biological process; therefore, some batches of vaccine may fail for reasons that are not always clear and ultimately it may lead to further delay in production^41^. There are quite few manufacturers in the world that can produce vaccines on a large enough scale to meet the needs of a pandemic. Pfizer, Moderna, and other pharmaceutical companies comprehensively addressed the challenges related to the manufacturing of vaccines faced by researchers and pharmaceutical experts.

#### Logistics and transportation issues

The shipment of the COVID-19 vaccines from the production point to the final retailers over a few days is a complex system due to many factors like storage factories, airplanes, cargo stations, warehouses and so on^42^. To avoid more waves of the COVID-19 pandemic, it is important to address all the issues related to the logistic, transportation and distribution of vaccines. According to a report by the logistic firm Boyle Transportation, it is estimated that 14 billion COVID-19 doses will be required for two doses per person on earth, which is itself a challenging task^43^. Considering the deployment of the COVID-19 vaccine, a transparent distribution and implementation are indispensable for vaccines. Successful distribution and deployment will result from the collaboration of complex networks of governing agencies, healthcare workers, the public and companies^44^.

#### Cold chain management (CCM) issues

During the current COVID-19 pandemic, the main challenge was to keep the integrity of vaccine. COVID-19 vaccine are temperature sensitive and they require ultra-care to be properly monitored during its shipping. The integrity of the vaccine is solely dependent on cold chain management. The most important part of any COVID-19 vaccine program is the cold chain. Infect, the success of the SCM process hinges on the correct implementation of the CCM. The efficacy of the vaccine is achieved by maintaining the cold chain process in the right way. The cold chain unit ensures that cold chain storage facilities are provided from the manufacturing to the administration site of the vaccine. According to a report released by the WHO, it is estimated that 2.8 million doses of vaccines were lost in 2011 in five countries due to the failure of the cold chain. Cold chain solitary contributes to 80% of all vaccination costs^45^. The most common temperature for storing vaccines ranges from 2 to 8 degrees but some of the coronavirus vaccines may require storage temperatures up to − 70° centigrade. Storing vaccines at up to low temperatures is a big challenge for cold chain storage infrastructure and it is ought to be addressed to keep the vaccine under thermal requirements.

#### Issues in vaccine administration

While administrating the corona vaccine, still there are some challenges that are required to be addressed to complete the entire cycle of SCM. These major issues include like lack of awareness, people reluctance to vaccine, lack of ancillary supplies etc.

The complete details of challenges and bottlenecks during each phase of the COVID-19 vaccine SCM process are given in Table 1^41,46–51^.

**Table 1:** COVID-19 vaccine SCM challenges.

S. N	SCM phase	Challenges and bottlenecks
1	Manufacturing	Quality assurance and quality control Production rate Shortage of biological material and technical kits for vaccine production Lack of components and ingredients such as lipids and plastic bags etc Variants of COVID-19 virus Potency testing/Sterility Scarcity of skilful personnel Failing in establishment of long-term outcomes based on clinical trials Delay in regulatory approvals Safety and efficacy of vaccines Construction and validation of vaccine development platforms Installing the latest manufacturing plants or upgrading the existing ones
2	Logistics and transportation	Cost and payer issues Lack of communication at state levels and shipping companies Improper or unfamiliar routing Lack of tracking and monitoring vaccines Privacy and security concerns People migration from one place to other Fraud, theft or loss or illegal access Limited funding Lack of planning Poor communication infrastructure Building shipping networks for new locations
3	Cold Chain management	Dry ice shortage Lack of trained staff Maintenance of required temperature Cost of ultra-cold storage boxes Lack of power supply in rural areas Lapses in regular temperature monitoring Need of manufacturing temperature stable vaccine
4	Administration	Public reluctance to vaccination Lack of awareness Covering large population Identifying the vulnerable people group Missing of ancillary supplies Disposable of medical equipment at non-medical vaccination sites Protecting issues of healthcare staff Misinformation about vaccine Urgency challenge

A full-pledged technological solution to handle the problems of vaccine logistics and transportation is indispensable. After identifying issues in the SCM phases, it is important to provide reasonable solutions towards these challenges and issues. The major focus of this research work is to provide IoT- based solutions by bringing the most viable IoT platform (discussed in next section).

After identifying the challenges, the next step was to select IoT platform alternatives. In this study, we selected the most popular IoT platforms for evaluation. In this study, we selected twenty (20) IoT platforms such as Carriots (P_1_), AWS IoT (P_2_), Azure IoT (P_3_), IBM Watson (P_4_), Bosh IoT suit (P_5_), Particle (P_6_), DeviceHive (P_7_), Xively (P_8_), Kaa(P_9_), Google cloud (P_10_), The Things (P_11_), thethings.iO (P_12_), Axeda (P_13_), Zetta (P_14_), Oracle IoT(P_15_), ThingsBoard (P_16_), Everything(P_17_), ThingSpeak (P_18_), ThingsWorx (P_19_) and Openremote (P_20_). All the IoT platforms selected in this study are based on the expert’s opinion and applications in industry for L&T.

After selecting alternatives, the next step of the proposed framework is to set the benchmark or criteria for selection of IoT platforms. In this step, we also selected the most famous and well-known IoT platforms for evaluation and ranking purpose. The criteria are designed based on the features/parameters of IoT platform. Therefore, we performed features-analysis to include the most relevant features that are covering all IoT business needs. The parameters selected from literature and they are consulted with the expert panel in IoT. The detail about the the finally selected parameters with the literature source is given in Table 2.

**Table 2:** Evaluation parameters description and literature sources.

Parameter	Ref	Description
Scalability (C_1_)	^34,35,52–58^	The potential of platform to support hardware and software
Availability (C_2_)	^35,59–61^	Platform ability to work in case of any failure or error is encountered
Security(C_3_)	^34–36,52,54,56,57,59,61–67^	Secure transmission, development of secure applications and protection against cyber-attacks
Cost/Price(C_4_)	^36,52,59,63,68–70^	Total price charged by the platform vendor
Device management (C_5_)	^33,36–38,52,63,67,71–73^	The ability of IoT platform to manage, control, authenticate and update the apps of IoT devices and assessing the related data
Deployment (C_6_)	^37,59,67,71,72,74^	It shows the IoT platform features-based development
Protocol support(C_7_)	^52,54,57,59,63^	Protocols list adopted by platform for different services
Usability(C_8_)	^52,75^	Platform easy development, tutorial and documentation
Interoperability (C_9_)	^57,63,66^	Integration with third party tools and hardware by using APIs

**Figure 3:**
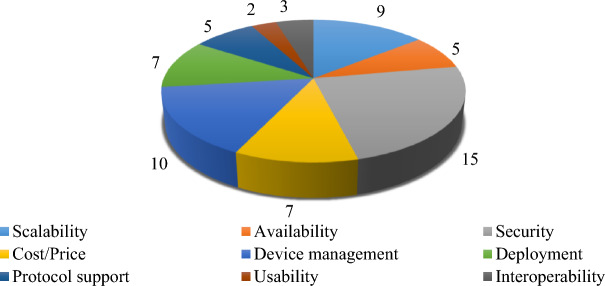
Frequency of feature citations.

These parameters are selected based on the frequency of occurrence in the existing literature. The complete detail about the number of times a particular feature of IoT platform used for evaluating the IoT platform is given in Fig. 3.

**Figure 4:**
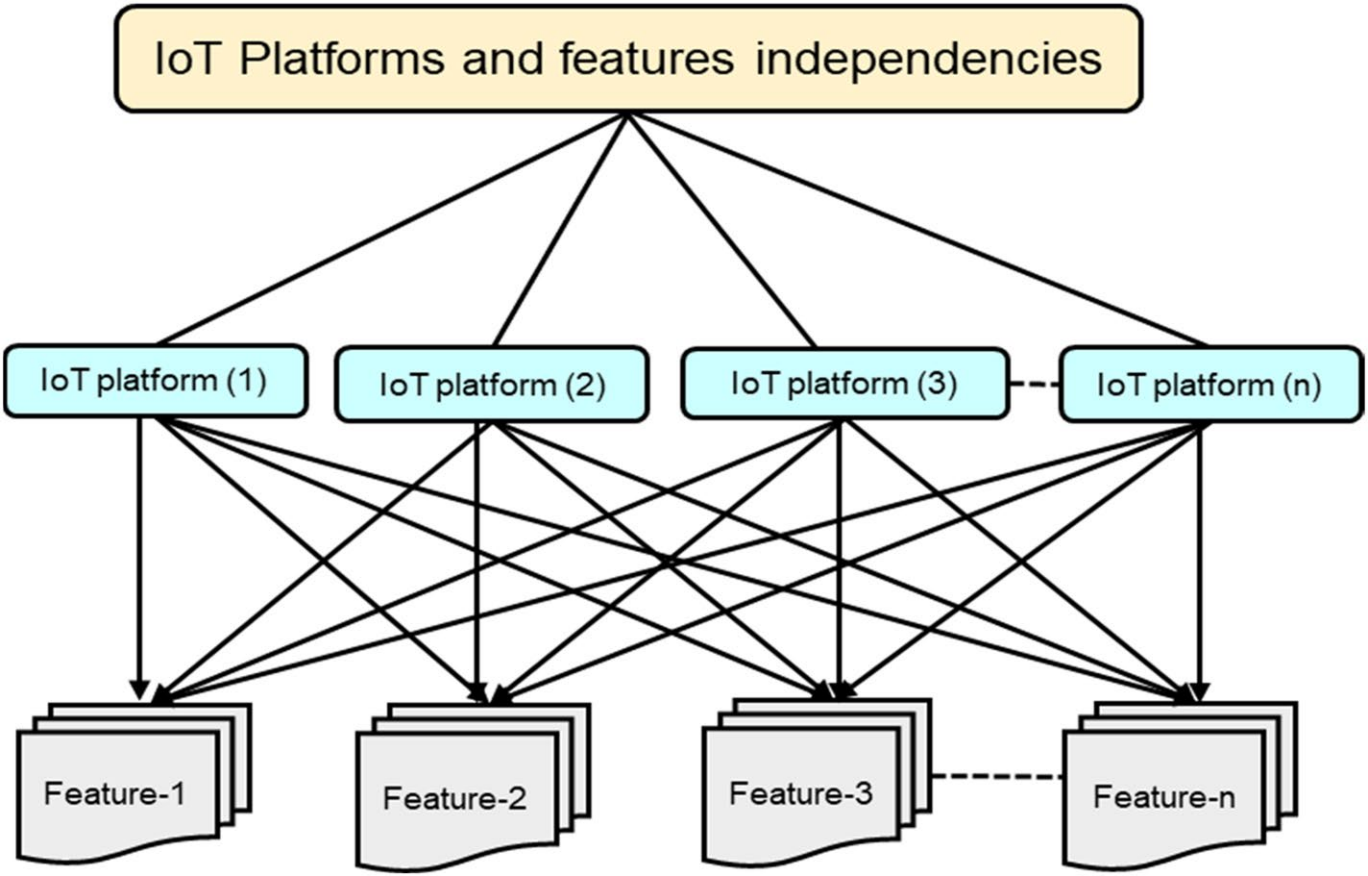
Hierarchy and inter-dependencies of features and IoT platforms.

For any platform, it is important to have certain features and any certain features is required to be supported by every possible IoT platform. Every platform is interdependent on every feature and vice versa. The hierarchical structure of “n” number of IoT platforms with “n” number of features are given in Fig. 4.

**Figure 5:**
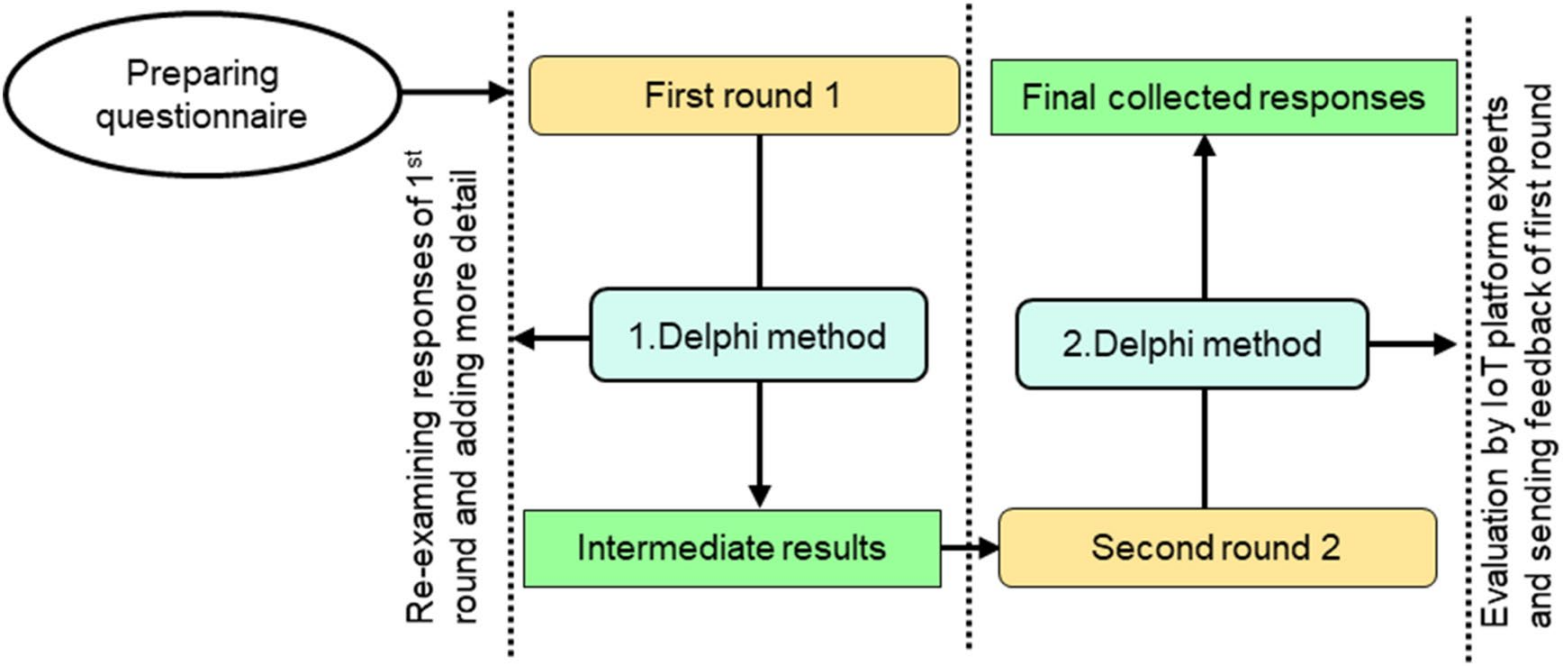
Estimate-Talk-Estimate technique for collecting expert’s response.

After finalizing IoT platforms alternatives we conducted a survey for collecting data. In this regard, a case study has been conducted for collecting data related to selected platforms and verifying the criteria features. In this cases study, the IoT platform vendors along with SCM experts were consulted in finalizing the list of important features. This case study followed a well-known method such as Estimate-Talk-Estimate technique which is also known as Delphi method. This technique is applied to check the criteria features by consulting the experts group in the field of IoT. They provided their valuable response about the features selected in this study. The responses or opinions from the expert’s panel are collected based on this technique. Intermediate results are obtained after applying ETE technique in first round. Second round completes the collection of data about the IoT platforms with respect to criteria. Our focus is to collect the most relevant data based on the issues (already discussed) in selection of IoT platforms. The complete detail of applying this technique in context of receiving responses from expert’s panel is given in Fig. 5.

During the survey the experts’ opinions were recorded about the selected platforms against the designed criteria. The expert groups were asked to provide response based on numeric values. They provided response about every platform with respect to criteria features based on Saaty’s scale which is ranging from 1 to 10. All the expert groups/participants provided informed consent for participation in this study. All the methods were carried out by following the relevant guidelines and regulations.

The outcome of the final activity after using ETE techniques is a tabular data consisted of IoT platform and features. This data will be provided as input in the decision matrix form in next step. Data related to decision matrix about the selected IoT platforms against the criteria feature is given in Table 3. The next step was to assign weights to criteria features using CRITIC and evaluate and prioritize IoT platform by using TOPSIS.

**Table 3:** Decision matrix.

Alt	C_1_	C_2_	C_3_	C_4_	C_5_	C_6_	C_7_	C_8_	C_9_
P_1_	6	7	5	8	4	3	9	7	8
P_2_	3	4	8	6	5	7	6	9	7
P_3_	9	7	8	5	7	7	6	4	5
P_4_	5	7	4	8	9	6	5	8	7
P_5_	7	5	3	8	9	6	4	2	6
P_6_	4	8	7	5	3	6	9	1	5
P_7_	8	5	7	3	2	8	6	2	8
P_8_	2	7	7	6	8	9	3	4	5
P_9_	7	6	3	3	8	8	9	5	4
P_10_	1	7	5	7	6	8	2	4	3
P_11_	3	5	8	6	4	3	6	8	8
P_12_	3	6	4	6	3	6	8	3	3
P_13_	3	5	5	3	6	2	5	6	7
P_14_	2	3	5	8	2	1	8	3	1
P_15_	3	3	5	6	4	7	6	8	8
P_16_	5	4	5	6	3	3	4	7	5
P_17_	3	6	5	4	6	6	2	4	7
P_18_	7	7	3	6	7	3	7	5	4
P_19_	4	4	3	6	7	8	8	6	3
P_20_	2	4	2	6	7	8	8	4	4

### Assigning weights to feature/parameters

The values assigned by expert panel during the case study may be suffering from biasness and subjectivity, therefore our focus is to avoid the element of biasness in our judgment. For this purpose, we applied the most famous MCDM method known as CRITIC. This method uses mathematical procedure and statistical approaches to solve any MCDM problem. The first step of CRITC method is to build a decision matrix. The data about the decision matrix (D_ij_) is derived from Table 4 as given below.

$${\text{D}}_{{{\text{ij}}}} { = }\left[ {\begin{array}{*{20}l} {} \hfill & {{\text{C}}_{{1}} } \hfill & {{\text{C}}_{2} } \hfill & {{\text{C}}_{3} } \hfill & {{\text{C}}_{4} } \hfill & {{\text{C}}_{5} } \hfill & {{\text{C}}_{6} } \hfill & {{\text{C}}_{7} } \hfill & {{\text{C}}_{8} } \hfill & {{\text{C}}_{9} } \hfill \\ {{\text{A}}_{{1}} } \hfill & 6 \hfill & 7 \hfill & 5 \hfill & 8 \hfill & 4 \hfill & 3 \hfill & 9 \hfill & 7 \hfill & 8 \hfill \\ {{\text{A}}_{2} } \hfill & 3 \hfill & 4 \hfill & 8 \hfill & 6 \hfill & 5 \hfill & 7 \hfill & 6 \hfill & 9 \hfill & 7 \hfill \\ {{\text{A}}_{3} } \hfill & 9 \hfill & 7 \hfill & 8 \hfill & 5 \hfill & 7 \hfill & 7 \hfill & 6 \hfill & 4 \hfill & 5 \hfill \\ {{\text{A}}_{4} } \hfill & 5 \hfill & 7 \hfill & 4 \hfill & 8 \hfill & 9 \hfill & 6 \hfill & 5 \hfill & 8 \hfill & 7 \hfill \\ {{\text{A}}_{5} } \hfill & 7 \hfill & 5 \hfill & 3 \hfill & 8 \hfill & 9 \hfill & 6 \hfill & 4 \hfill & 2 \hfill & 6 \hfill \\ {{\text{A}}_{6} } \hfill & 6 \hfill & 1 \hfill & 8 \hfill & 5 \hfill & 5 \hfill & 1 \hfill & 4 \hfill & 1 \hfill & 7 \hfill \\ {{\text{A}}_{7} } \hfill & 4 \hfill & 1 \hfill & 6 \hfill & 6 \hfill & 2 \hfill & 1 \hfill & 3 \hfill & 2 \hfill & 8 \hfill \\ {{\text{A}}_{8} } \hfill & 7 \hfill & 1 \hfill & 6 \hfill & 8 \hfill & 1 \hfill & 1 \hfill & 1 \hfill & 1 \hfill & 8 \hfill \\ {{\text{A}}_{9} } \hfill & 7 \hfill & 1 \hfill & 5 \hfill & 8 \hfill & 1 \hfill & 1 \hfill & 5 \hfill & 1 \hfill & 7 \hfill \\ {{\text{A}}_{10} } \hfill & 5 \hfill & 1 \hfill & 7 \hfill & 7 \hfill & 6 \hfill & 1 \hfill & 2 \hfill & 1 \hfill & 5 \hfill \\ {{\text{A}}_{11} } \hfill & 6 \hfill & 1 \hfill & 5 \hfill & 6 \hfill & 7 \hfill & 1 \hfill & 1 \hfill & 1 \hfill & 4 \hfill \\ {{\text{A}}_{12} } \hfill & 6 \hfill & 1 \hfill & 7 \hfill & 8 \hfill & 7 \hfill & 1 \hfill & 1 \hfill & 1 \hfill & 4 \hfill \\ {{\text{A}}_{{{13}}} } \hfill & 8 \hfill & 1 \hfill & 5 \hfill & 6 \hfill & 5 \hfill & 1 \hfill & 5 \hfill & 1 \hfill & 7 \hfill \\ {{\text{A}}_{{{14}}} } \hfill & 7 \hfill & 7 \hfill & 8 \hfill & 8 \hfill & 1 \hfill & 7 \hfill & 8 \hfill & 8 \hfill & 5 \hfill \\ {{\text{A}}_{{{15}}} } \hfill & 5 \hfill & 8 \hfill & 6 \hfill & 6 \hfill & 8 \hfill & 1 \hfill & 1 \hfill & 1 \hfill & 7 \hfill \\ {{\text{A}}_{{{16}}} } \hfill & 5 \hfill & 1 \hfill & 4 \hfill & 6 \hfill & 5 \hfill & 1 \hfill & 1 \hfill & 1 \hfill & 5 \hfill \\ {{\text{A}}_{{{17}}} } \hfill & 5 \hfill & 1 \hfill & 6 \hfill & 4 \hfill & 6 \hfill & 1 \hfill & 2 \hfill & 1 \hfill & 5 \hfill \\ {{\text{A}}_{{{18}}} } \hfill & 7 \hfill & 1 \hfill & 5 \hfill & 6 \hfill & 7 \hfill & 1 \hfill & 1 \hfill & 1 \hfill & 7 \hfill \\ {{\text{A}}_{{{19}}} } \hfill & 5 \hfill & 1 \hfill & 6 \hfill & 8 \hfill & 7 \hfill & 1 \hfill & 7 \hfill & 1 \hfill & 7 \hfill \\ {{\text{A}}_{{{20}}} } \hfill & 5 \hfill & 1 \hfill & 4 \hfill & 6 \hfill & 7 \hfill & 1 \hfill & 1 \hfill & 1 \hfill & 6 \hfill \\ \end{array} } \right]$$

**Table 4:** Criteria weights.

Criteria	Standard deviation	Correlation	Weight
Scalability	0.458	2.506	0.172
Availability	0.487	1.189	0.082
Security	0.584	1.518	0.104
Cost	0.672	1.693	0.117
Device management	0.565	1.471	0.101
Deployment	0.497	1.696	0.117
Protocol support	0.671	1.693	0.117
Usability	0.709	1.495	0.098
Interoperability	0.659	1.344	0.092

The input provided to CRITIC method is a decision matrix, where mathematical calculations are performed to obtain weights to the features as shown in Table 4.

**Figure 6:**
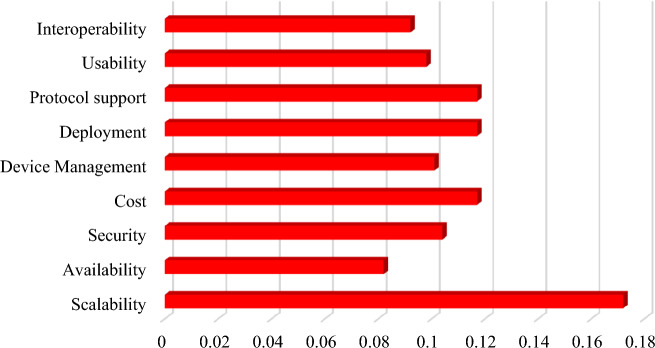
Features weights.

The output obtained from the CRITIC method indicates that the highest weightage is assigned to scalability feature, followed by cost, deployment and protocol with equal numeric value of weights. The complete detail of weights assigned to the features is visually depicted in Fig. 6.

### IoT platforms evaluation and prioritization

The CRITIC method has been applied for assigning weights to the features of criteria. After giving weights to the features of IoT platform, next step is to evaluate and rank IoT platform based on the designed criteria features. TOPSIS method has been applied to evaluate the platform alternatives based on criteria. The stepwise detail of TOPSIS method is given below as.

#### Making decision matrix

In first step of TOPSIS method the decision matrix is formed with “n” number of criteria and alternatives. The decision matrix denoted by “D” is built by using Eq. (1). Decision matrix is normally built with the assistance of expert groups.

1 $${\text{D}} = \begin{array}{*{20}c} {{\text{A}}_{1} } \\ {\begin{array}{*{20}c} \vdots \\ A \\ \end{array}_{{\text{n}}} } \\ \end{array} \left[ {\begin{array}{*{20}c} {{\text{C}}_{1} } & { \ldots \ldots \ldots .. \ldots \ldots ....} & {{\text{C}}_{{\text{n}}} } \\ {{\text{X}}_{11} } & { \ldots \ldots \ldots \ldots ..} & {{\text{X}}_{{1{\text{n}}}} } \\ { } & { } & { } \\ \vdots & \ddots & \vdots \\ {{\text{X}}_{{{\text{m}}1}} } & { \ldots \ldots \ldots \ldots \ldots \ldots } & {{\text{X}}_{{{\text{mn}}}} } \\ \end{array} } \right]$$

In this matrix A_1,_ A_2,_ A_3_…A_n,_ denote the alternatives and C_1,_ C_2,_ C_3_…C_n_ show the criteria involved in decision making.

#### Building normalized decision matrix

The data given in decision matrix (*D)* originates from different sources, therefore, it has to be normalized by converting into a dimensionless matrix. The comparison of different criteria is done via dimension matrix. A normalized decision matrix (R_ij_) is built by using the following Eq. (2). To negate the element of the biasness the decision matrix is normalized.

2 $${\text{R}}_{{{\text{ij}}}} = \frac{{{\text{X}}_{{{\text{ij}}}} }}{{\sqrt {\mathop \sum \nolimits_{{{\text{i}} = 1}}^{{\text{m}}} {\text{x}}_{{{\text{ij}}}}^{2} } { }}}$$

For i = 1…m and j = 1…n.

##### Step-3. Determining the weighted normalized decision matrix

Sometimes, all attributes may not be same values or importance. In this regard, “V” is calculated which indicates the values of weighted normalized decision matrix. It can be computed by the multiplication of every element (R_ij_) of normized decision matrix with a random weight number as follows using Eq. (3).

3 $$\begin{gathered} {\text{V}} = {\text{V}}_{{{\text{ij}}}} = {\text{W}}_{{\text{j}}} \times {\text{ R}}_{{{\text{ij}}}} { } \hfill \\ {\text{V = }}\left[ {\begin{array}{*{20}c} {{\text{V}}_{{{11}}} } & {{\text{V}}_{{{12}}} } & {{\text{V}}_{{{\text{1j}}}} } & {{\text{V}}_{{{\text{1n}}}} } \\ \vdots & \vdots & \vdots & \vdots \\ \vdots & \vdots & \vdots & \vdots \\ {{\text{V}}_{{{\text{i1}}}} } & {{\text{V}}_{{{\text{i2}}}} } & {{\text{V}}_{{{\text{ij}}}} } & {{\text{V}}_{{{\text{in}}}} } \\ \vdots & \vdots & \vdots & \vdots \\ \vdots & \vdots & \vdots & \vdots \\ {{\text{V}}_{{{\text{m1}}}} } & {{\text{V}}_{{{\text{m2}}}} } & {{\text{V}}_{{{\text{mi}}}} } & {{\text{V}}_{{{\text{mn}}}} } \\ \end{array} } \right]{ = }\left[ {\begin{array}{*{20}c} {{\text{w}}_{{1}} {\text{r}}_{{{11}}} } & {{\text{w}}_{{1}} {\text{r}}_{{{11}}} } & {{\text{w}}_{{1}} {\text{r}}_{{{11}}} } & {{\text{w}}_{{1}} {\text{r}}_{{{11}}} } \\ \vdots & \vdots & \vdots & \vdots \\ \vdots & \vdots & \vdots & \vdots \\ {{\text{w}}_{{1}} {\text{r}}_{{{11}}} } & {{\text{w}}_{{1}} {\text{r}}_{{{11}}} } & {{\text{w}}_{{1}} {\text{r}}_{{{11}}} } & {{\text{w}}_{{1}} {\text{r}}_{{{11}}} } \\ \vdots & \vdots & \vdots & \vdots \\ \vdots & \vdots & \vdots & \vdots \\ {{\text{w}}_{{1}} {\text{r}}_{{{11}}} } & {{\text{w}}_{{1}} {\text{r}}_{{{11}}} } & {{\text{w}}_{{1}} {\text{r}}_{{{11}}} } & {{\text{w}}_{{1}} {\text{r}}_{{{11}}} } \\ \end{array} } \right] \hfill \\ \end{gathered}$$

##### Step-4. Finding ideal positive and negative solutions

**Figure 7:**
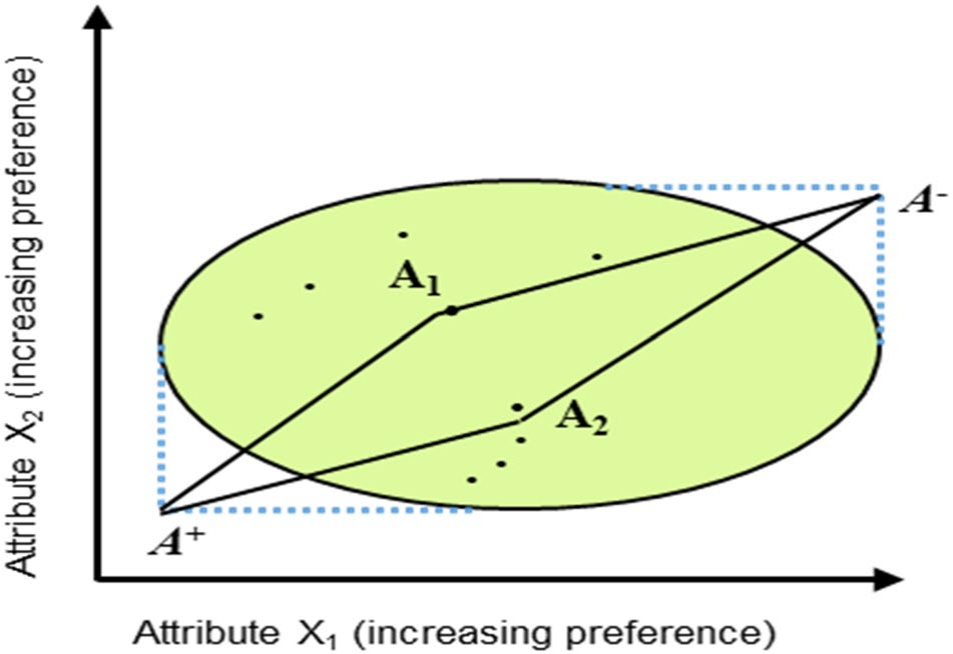
Ideal positive and ideal negative points.

The positive ideal solutions represented by A^+^ and negative eal solutions denoted by A^−^ are determined by using previously calculated weighted decision matrix. They are found by using the following Eqs. (4) and (5) respectively.

4 $${\text{ A}}^{ + } = \left\{ {{\text{V}}_{1}^{ + } ,{\text{ V}}_{2}^{ + } ,{\text{ V}}_{3}^{ + } ,{\text{ V}}_{{\text{n}}} { }} \right\}{\text{ Where V}}_{{\text{j}}}^{ + } {\text{ is }}\left\{ {\left( {{\text{max}}\left( {{\text{V}}_{{{\text{ij}}}} } \right){\text{ifj}} \in {\text{J}}} \right),\left( {{\text{min V}}_{{{\text{ij}}}} {\text{ if j}} \in {\text{J}}^{\prime } } \right)} \right\}$$

5 $${\text{A}}^{ - } = \left\{ {{\text{V}}_{1}^{ - } ,{\text{ V}}_{2}^{ - } ,{\text{ V}}_{3}^{ - } ,{\text{ V}}_{{\text{n}}}^{ - } } \right\},{\text{ Where V}}_{{\text{j}}}^{ - } {\text{ is }}\left\{ {{\text{mini}}\left( {{\text{V}}_{{{\text{ij}}}} } \right){\text{if j}} \in {\text{J}});\left( {{\text{maxi V}}_{{{\text{ij}}}} {\text{ if j}} \in {\text{J}}^{\prime } } \right)} \right\}$$

where “J” denotes the beneficial attributes and “J' “ is shows non-beneficial attributes. For complete understanding ideal positive (A^+^) and ideal negative point (A^−^), the visual representation is given in Fig. 7^76^.

##### Step-5. Determining the separation measures

Ideal separation measure is denoted by S^+^ and no-ideal separation measure is represented by S^−^ which are obtained with the help of using the following two mathematical equations.

6 $$S^{ + } = \sqrt {\mathop \sum \limits_{{{\text{J}} = 1}}^{{\text{n}}} ({\text{V}}_{{{\text{ij}}}} - {\text{V}}^{ + } )^{2} { }} {\text{ For i}} = 1 \ldots {\text{ m }}$$

7 $$S^{ - } = \sqrt {\mathop \sum \limits_{{{\text{J}} = 1}}^{{\text{n}}} ({\text{V}}_{{{\text{ij}}}} - {\text{V}}^{ - } )^{2} { }} {\text{For i}} = 1 \ldots {\text{ m }}$$

##### Step-6. Finding of relative closeness

The relative closeness symbolized by C_i_ variable is impoant for the purpose of final ranking and it is found with the help of the Eq. (8) as given below.

8 $${\text{ C}}_{{\text{i}}} = \frac{{{\text{S}}_{{{\text{i}}^{ - } }} }}{{\left( {{\text{S}}_{{\text{i}}}^{ + } + {\text{S}}_{{\text{i}}}^{ - } } \right)}}{ }0 \le {\text{C}}_{{\text{i}}} \le 1{ }$$

##### Step-7. Ranking of alternatives

The alternatives were ranked by using the value of C_i_ i.e. the alternative with higher value of C_i_ value stands higher in ranking and performance and alternative having low value of C_i_ is considered as low in performance and significance. Ranking of alternatives or preferences can be done in both descending and ascending order.

**Figure 8:**
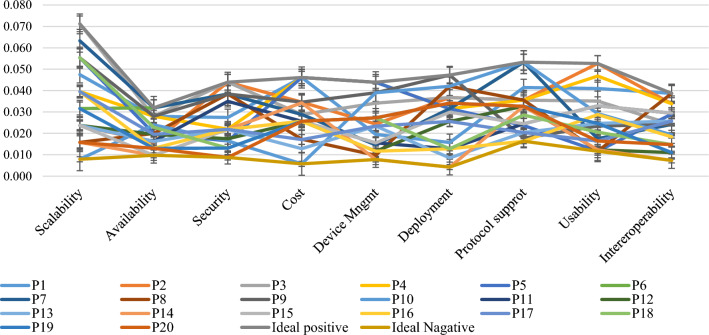
Comparison of IoT platforms alternatives based on A^+^ and A^−^.

After assigning weights to the features, the TOPSIS approach is applied to rank the IoT platforms based on the criteria by performing mathematical and statistical calculation. The application of TOPSIS produces numeric results that can be used for ranking of IoT platforms. TOPSIS method uses a sequential procedure for ranking the IoT platform alternatives. It ranks the IoT alternatives based on the values of performance index or relative closeness (C_i_). The higher value of C_i_ indicates that alternative may be considered as best choice among the comparing list of alternatives. The decision matrix already obtained is provided as input to the TOPSIS. This method uses Eq. (3) for converting decision matrix to normalized form. The major purpose of this procedure is to convert the data into a form that is free from personal biasness. Then, Eqs. (4) and (5) have been applied to obtain ideal positive and ideal negative solutions. The detail of all IoT platform alternatives against the features with respect to ideal positive and ideal negative solutions is given in Fig. 8.

All the values of every platform against the criteria are between ideal positive and ideal negative solutions. Hence, our results are accurate and we can proceed to the next step. The separation measures (S^+^, S^−^) and relative closeness (C_i_) are calculated by using Eqs. (6), (7) and (8) respectively and output is given in Table 5. The value of C_i_ is i5mportant consideration as it decides the outcome of the evaluation process i.e. the higher value of C_i_ indicates the best solution and lower value shows the worst cases. According to the results of Table 5, P3 alternative has the highest value of all the IoT platforms.

**Table 5:** Platform alternatives ranking.

Platform	S_+_	S_-_	S_(+)_ + S_-_	C_i_	Rank
P_1_	0.052	0.081	0.133	0.6094	3
P_2_	0.059	0.080	0.139	0.5766	5
P_3_	0.037	0.095	0.132	0.7211	1
P_4_	0.086	0.086	0.173	0.5000	7
P_5_	0.064	0.081	0.145	0.5612	6
P_6_	0.067	0.070	0.137	0.5133	8
P_7_	0.054	0.086	0.140	0.6141	4
P_8_	0.085	0.063	0.148	0.4248	11
P_9_	0.053	0.087	0.140	0.6222	2
P_10_	0.086	0.067	0.153	0.4372	10
P_11_	0.079	0.050	0.129	0.3874	15
P_12_	0.089	0.040	0.128	0.3092	18
P_13_	0.090	0.036	0.126	0.2867	19
P_14_	0.102	0.036	0.138	0.2625	20
P_15_	0.078	0.050	0.128	0.3930	13
P_16_	0.083	0.046	0.128	0.3556	16
P_17_	0.077	0.050	0.127	0.3924	14
P_18_	0.073	0.060	0.133	0.4491	9
P_19_	0.076	0.052	0.128	0.4079	12
P_20_	0.089	0.046	0.134	0.3403	17

**Figure 9:**
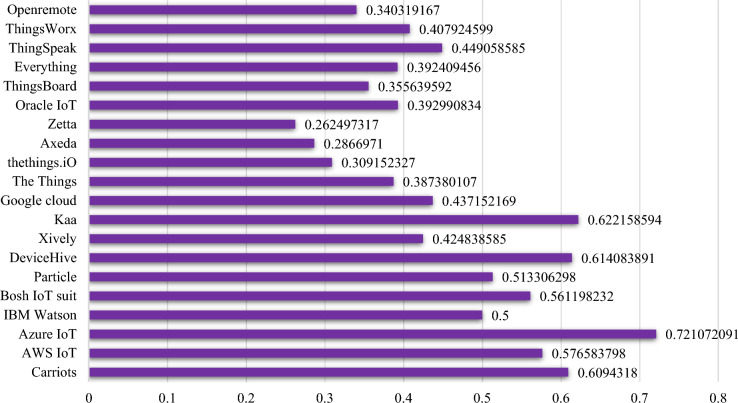
Platforms consistency index value.

So, Microsoft Azure (P3) is reckoned as best choice among the list of IoT platforms based on our proposed criteria. The comparison of all IoT platforms selected in this study is given in Fig. 9.

**Figure 10:**
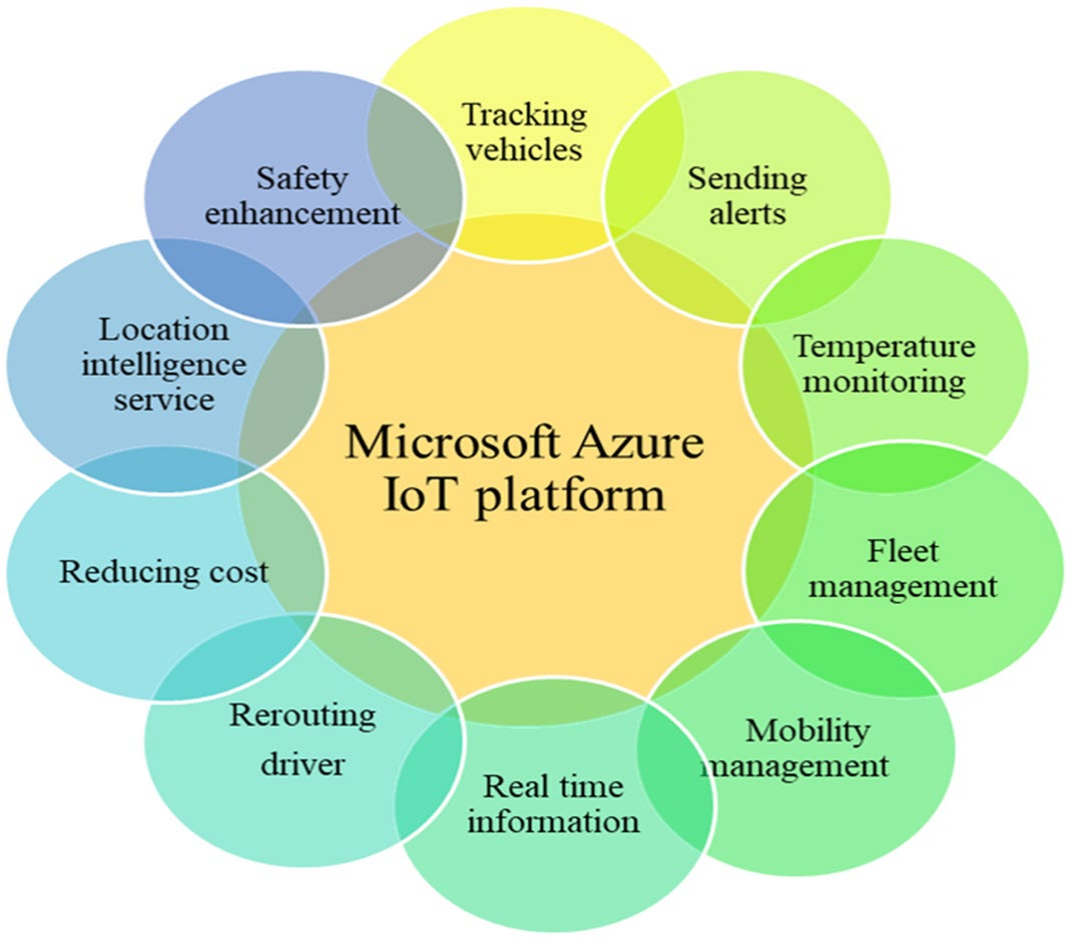
Microsoft Azure IoT platform features of logistics and transportation.

Thus, the finding of this study suggests that Microsoft Azure IoT platform is the right candidate for shipping of COVID-19 vaccine. It has the ability to support multiple features in respect of supply chain operation and logistics process. This platform uses different technologies such as Azure cloud, Azure IoT hub, Azure machine learning and Azure maps to provide smart delivery in L&T. Microsoft Azure IoT provides smart transportation infrastructure, assessing the road conditions, real time and historic traffic management. It uses real time data to bring fleet operation or smart logistics by sending alerts, monitoring performance, optimizing the delivery routes and responding to the delay as they occur. In case of any issue or delay encounters it has the potential to quickly analyze the problem and bring predictive maintenance. It is powered by Azure map to provide the smart logistics and fleet management options such as vehicle monitoring, arrival time, vehicle’s route, checking humidity level etc. The complete summary of features supported by Azure IoT platform in L&T are given in Fig. 10^77^. Hence, the adoption of Microsoft Azure IoT is reckoned as the best IoT-driven solution for the shipping and logistics of COVID-19 vaccine in this pandemic.

## Framework evaluation and testing

It is important to evaluate and test the IoT evaluation framework of IoT platforms. The proposed evaluation framework is checked by the experts for the features included and excluded during the criteria designing. To achieve this, we conducted a case study, in which expert participated and they presented their opinions about the number of features and gravity of features of IoT platforms selected for evaluation. We evaluated framework by two different approaches such as evaluation by experts i.e. checking the criteria feature and survey based evaluation which is focused on assessing the overall performance of proposed framework. The detail of both assessing methods is given as.

### Evaluation by experts

After finalizing and building the evaluation framework, we evaluated the features of the proposed framework by consulting the experts group due to the theoretical nature of framework. This framework is evaluated by three evaluation parameters such as accuracy, precision and recall. As, said earlier that the features are the building block of IoT platform. Therefore, it is important to check relevant, irrelevant, recommend and not recommend features to keep the framework working well and producing the desired outcomes. For evaluation purpose, we used four type of variables to classify the features such as the number of features recommend by our experts group and the proposed framework is denoted by “a”. The number features only suggested only by proposed framework are represented by variable “b”. The number of features only suggested by expert panel is shown by “c”. Similarly, the variable “d” indicates the number of features not suggested by evaluation framework and nor suggested by expert group. This method used for evaluation and testing of evaluation framework is well-known technique often used to evaluate the context-base recommendations systems^78,79^. The evaluation parameters such as accuracy, precision and recall are obtained by using the following Eqs. (9), (10) and (11) respectively.

9 $${\text{Accuracy}} = {\raise0.7ex\hbox{${\left( {{\text{a}} + {\text{d}}} \right)}$} \!\mathord{\left/ {\vphantom {{\left( {{\text{a}} + {\text{d}}} \right)} {\left( {{\text{a}} + {\text{b}} + {\text{c}} + {\text{d}}} \right)}}}\right.\kern-0pt} \!\lower0.7ex\hbox{${\left( {{\text{a}} + {\text{b}} + {\text{c}} + {\text{d}}} \right)}$}}$$

10 $${\text{Precision}} = {\raise0.7ex\hbox{${\left( {\text{a}} \right)}$} \!\mathord{\left/ {\vphantom {{\left( {\text{a}} \right)} {\left( {{\text{a}} + {\text{b}}} \right)}}}\right.\kern-0pt} \!\lower0.7ex\hbox{${\left( {{\text{a}} + {\text{b}}} \right)}$}}{ }$$

11 $${\text{Recall}} = {\raise0.7ex\hbox{${\left( {\text{a}} \right)}$} \!\mathord{\left/ {\vphantom {{\left( {\text{a}} \right)} {\left( {{\text{a}} + {\text{c}}} \right)}}}\right.\kern-0pt} \!\lower0.7ex\hbox{${\left( {{\text{a}} + {\text{c}}} \right)}$}}{ }$$

The feature classification in terms of recommended, not-recommended, relevant and irrelevant is given in Table 6.

**Table 6:** Recommendations classifition.

	Relevant	Irrelevant
Recommended	a	c
Not recommended	b	d

The complete process of finding the evaluation parameters expert panel in comparison to our evaluation framework is given in Table 7.

**Table 7:** Recommendation evaluation parameters results.

Experts	a	b	c	d	Accuracy (%)	Recall (%)	Precision (%)
1	9	1	1	13	92	90	90
2	29	0	1	19	98	100	97
3	13	1	1	13	93	93	93
4	12	0	1	7	95	100	92
5	13	1	2	11	89	93	87
6	26	0	2	9	95	100	93
7	12	2	0	9	91	86	100
8	11	1	1	16	93	92	92
9	13	1	1	12	93	93	93
10	26	2	1	8	92	93	96
Average					93%	94%	93%

### Survey based evaluation

Our survey based evaluation in this study is inspired from the study conducted in^78^. In this step to evaluate the IoT platform evaluation framework is through conducting a survey. In this survey, three expert group participated who are currently enrolled in Phd and MS degree programs. The framework evaluation through survey is conducted by using a five-points scale. In this survey, we asked 28 research questions of different categories and the responses are noted. The average of response in each category is calculated. The responses were collected based on the values assigned by the survey participants. The transforming of linguistic terms into numeric values based on five-point scale is given in Table 8.

**Table 8:** Survey scale description.

Numeric value	Linguistic Description
1	Strongly disagreed
2	Disagreed
3	Neutral
4	Agreed
5	Strongly agreed

We tested the proposed IoT evaluation framework based on a questionnaire consisted of many questions related to evaluation parameters such as security, usability, effectiveness and information and knowledge. The complete detail of questions/parameters asked during the survey and response of each group in terms of numeric values are given in Table 9.

**Table 9:** Responses of expert’s groups.

Evaluation parameters		Expert groups		
Security		Group 1	Group 2	Group 3
1	Framework includes the security aspects of SCM	4.1	3.5	4.5
2	Framework has the ability to adopt new security methods	4	4	3.8
3	The secure data transmission is addressed by framework	4	4.1	4.2
4	It assists in building secure IoT platform	4.2	4	4.3
5	The security evaluation mechanism is enough sufficient	3.8	4.2	4.5
6	It will help in selecting the right secure IoT solution in logistics & transportation system	4	3.8	3.8
Average		4.1	3.9	3.9

Usability				
7	The proposed framework is applicable or implementable	4.1	3.8	4
8	It supporting all the pre-requisites of IoT platform	3.7	4	4.2
9	The evaluation model of proposed framework is user-friendly	4.5	4.3	4.1
10	This platform is flexible and can be used in logistic & transportation domain	4	3.5	4
11	Framework is focusing on the user-experience	4.1	4.2	4.3
Average		4.0	3.9	4.1

Information and knowledge				
12	The criteria is balanced such that focusing on platform specific features and customer-specific features	3.5	4	4.4
13	The feedback obtained from evaluation framework can be used to enhance the existing platforms	3.9	4.1	4.4
14	It is providing the exact information about the IoT platforms in this area	4.4	4.2	4
15	It is educating the end-users for choosing the right platform	4.7	4.5	4.3
Average		4.1	4.1	4.2

Effectiveness				
16	The empirical results of proposed framework are correct	4.1	4.7	4.5
17	This platform produces consistent output	4.2	4.4	4.5
18	The framework will help the fleet managers to improve the business level by selecting sophisticated IoT platform	4.2	4.6	4.7
19	The relevant and updated list of features are included	4.1	3.7	4.2
20	The decision making methods are up to date	4.4	4.6	4.8
21	The criteria designed can be applied as benchmark for future use	4.2	4.1	3.9
22	Any important features is skipped, overlooked and repeated in criteria	4	4.5	4.1
23	The selected features are obtained from authentic source and cross checked	4.5	4	4.3
24	The identified issues are up to date and presented in detailed fashion	4	4.1	3.9
25	The framework is solving the problems of L&T sectors in true sense or not?	4.7	4.7	4.8
26	The evaluation framework validation method is up to date	4.4	4.3	4.7
27	Framework is effective in upgrading the security of solutions	4	3.7	3.5
28	It will help in achieving the goals of shipping companies in context of vaccine logistics delivery	3.4	3.3	3.6
Average		4.1	4.2	4.2
Accumulative average		4.07	4.02	4.10

After collecting responses from the expert’s groups in Table 8, it has become quite clear that mean values of all groups are more than 4. It means that all the expert groups are satisfied with our proposed IoT decision making model. All the group members have shown positive feedback and are in favour of recommending this evaluation framework for choosing the right IoT platform selection for L&T of COVID-19 vaccine.

## Proposed work validation

The survey based testing and verification were further validated by using a well-known MCDM techniques known as SAW method. This method has been applied to check the consistency and accuracy of our proposed decision making model. This method is very simple method. This method works based on the following major steps^80^.

- Step-1. Data normalization.
  The following equation is used to achieve the normalization of data.
  12 $$r_{ij} = \left\{ {\begin{array}{*{20}c} {x_{j}^{ - } /x_{ij} ,j\varepsilon \Omega min} \\ {x_{ij} /x_{j}^{ + } ,j\varepsilon \Omega max} \\ \end{array} } \right\}$$
- Step-2. Weight assignment to each criterion
  13 $$W = \left[ {w_{1} , w_{2} \ldots \ldots w_{n} } \right]$$
- Step-3. Ranking score calculation
  14 $$S_{i} = \mathop \sum \limits_{j = 1}^{M} w_{j} r_{ij} for i = 1,2...N$$
  where *S*_*i*_ Ranking score value for ith alternative, *W*_*j*_ Denotes the weight of jth criteria, *N* Shows number of alternatives, *M* Number of criteria, *r*_*ij*_ It is normalized rating, *x*_*ij*_ Denotes elements of decision matrix

As we have already computed the criteria weights by using CRITIC method so we skipped the first two steps in this technique. Equation (14) is used to calculate the ranking score for each IoT platform alternative. The results calculated through SAW approach in comparison to our proposed methodology are given in Table 10.

**Table 10:** Proposed work comparison with SAW technique.

SAW technique			Proposed work	
Alt(s)	S_i_	Ranking	C_i_	Ranking
P_1_	6.292	2	0.6094	3
P_2_	6.103	4	0.5766	5
P_3_	6.61	1	0.7211	1
P_4_	6.575	3	0.5000	7
P_5_	5.824	7	0.5612	6
P6	5.083	13	0.5133	8
P7	5.845	5	0.6141	4
P8	5.579	10	0.4248	11
P9	5.837	6	0.6222	2
P10	4.478	18	0.4372	10
P11	5.678	9	0.3874	15
P12	4.309	19	0.3092	18
P13	4.809	16	0.2867	19
P14	3.115	20	0.2625	20
P15	5.755	8	0.3930	13
P16	4.62	17	0.3556	16
P17	5.014	14	0.3924	14
P18	5.266	11	0.4491	9
P19	5.231	12	0.4079	12
P20	4.893	15	0.3403	17

**Figure 11:**
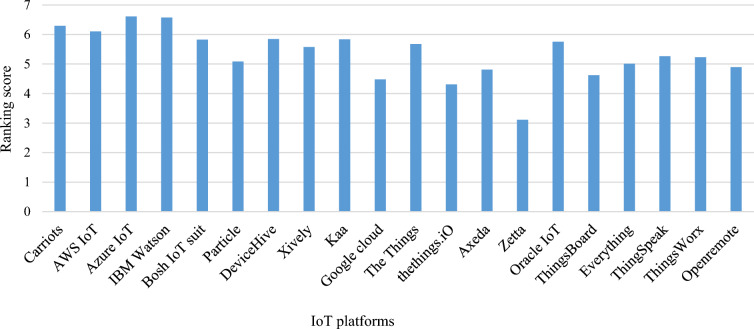
IoT platform ranking score by SAW method.

The results obtained after the application of SAW approach are also indicating that P_3_ platform has the higher value of ranking score in the list of selected IoT platforms. So, it is concluded from this empirical data that quantitative results are accurate and consistent. Hence, the proposed decision making model for IoT platform is also validated. The comparison of all selected IoT platforms based on the ranking score calculated by using SAW approach is given in Fig. 11. According to SAW approach, Microsoft Azure IoT platform stands on the top of all selected platform.

## Managerial implications

In this section, we are highlighting the recommendation and implications of the impacts of the proposed decision making model and selection of right IoT platform in logistics and transportation of COVID-19 vaccine from the perspective of managers and IoT platform developers. The proposed decision making model will enable the supply chain, cold chain, fleet, transportation and logistics managers to adopt the important aspects of IoT adoption or technology as whole or in incremental shape. As, the selection of IoT platform has influential impacts on the business operations of supply chain managers. Managers can adopt IoT opportunities to address bottlenecks in the conventional L&T vaccine delivery. The right selection of IoT platform helps managers to shift the product safer and efficient. IoT solutions help managers track the real-time visibility and traceability of vaccines by using various IoT sensors. These sensors are placed on pallets and have the ability to read light, temperature, humidity and other manufacturing details of the vaccine by sending alerts from the manufacturing point until the last mile logistics delivery. According to finding of this study, the selected IoT platform i.e. Microsoft Azure IoT provides features like reliability, efficiency, location intelligence, enhanced safety and low cost delivery through smarter ways.

The results of the presented decision making model provide a better and deeper understanding about the features selection and their importance. This study introduces the most important features about IoT technology adoption in L&T and helps in evaluating features based on their relative importance. The evaluation criteria feature defined in this research work is the most compact and cover all the dimensions of IoT platforms. Thus, this framework will help the L&T managers not to get worried about the features selection of IoT platform. For example, the device management feature of IoT platform allows the supply managers to take in to account the devices, sensors and actuators before applying them as business solution. According to the finding of our study the selected IoT platform such as Microsoft Azure IoT platform allows the IoT sensors management that provides geolocation services which means the cargo or vehicles or other assets can be monitored over a wide geographical distance in harsh environments. The selected IoT platform by this model also provides fleet management such as vehicle and driver and rout information.

The proposed decision making model will enable the fleet and supply managers to include the most critical features related to cold chain management as the COVID-19 vaccines are temperature sensitive. Therefore, the evaluation and decision making of IoT platform is imperative due to the significance and complexity of cold chain management. The deployment of right IoT platform will enable the supply chain managers to keep the track of temperature of COVID-19 vaccine in the route and to keep vital information about the real time status of vaccine shipping.

The supply chain managers are not the technical people in terms of computer literacy. Therefore, the selection of right IoT platform vendor requires more technical and expertise in this domain. Similarly, the number of IoT platform are drastically changing and the emergence of these platforms become a major issue for SCM managers to go through the extensive decision making process and selection the more suitable IoT platform. This evaluation framework is helpful not only for supply chain managers to deploy the best IoT platform for their business requirements but will also help the platform developers to develop, customize and integrate the most important features especially related to the shipping and logistics. It will also help the developers to incorporate all the features and functionalities based on their relative importance in IoT platforms. This decision making model platform will help them to update the IoT platform or customize it to provide up-to date services and features related to the L&T system.

In nutshell, the proposed decision making model will enable all the stakeholders working in logistics, transportation, supply chain, fleet; government agencies and platform developers to bring the advancement in the successful adoption of IoT technology for COVID-19 vaccine delivery to meet the emerging requirements of market.

## Study limitations

The proposed methodology produces promising results, covering all features and enhancing the decision making process but still are some limitations that may affect the practicability and stability of the proposed model as given.

- The criteria elements or parameters selected in this study are not absolute but are relative. It means that it is not mandatory that other models will also follow the selected features in their evaluation methodology. However, these are the best possible features supported by IoT platform with respect to deploying IoT technology for COVID-19 vaccine logistics and shipping.
- This methodology is consisted of TOPSIS, CRITIC and SAW approaches for evaluation purpose but still we believe that the proposed model can enhanced in by using the concept of fuzzy algorithms.
- The proposed model is the first time presented so authors feel that as more models will introduce in this domain then the proposed model can be better compared with the results. This is the main reason that we validated the proposed model using two different approaches such as survey-based evaluation and using SAW techniques to verify the quantitative results yielded by this model.

## Conclusion

The COVID-19 vaccine delivery from the point of manufacturing till the last mile delivery can be challenging job due to many factors involved such as cold storage management, safe delivery, quick delivery, temperature monitoring, improper routing etc. Different technological solutions have been suggested but the role of IoT is dominant. The success and operation of IoT hinges around the deployment of right IoT platform. Therefore, many IoT platforms have been introduced in the market. This proliferation resulted in decision making problems for SCM managers, who have less knowledge or expertise about the technology deployment and selection of right technology to perform various tasks easily and efficiently. As, there is no mechanism to compare directly the features of various IoT platforms due to the IoT platforms evolution and number of features supported. The first step of the proposed model is to define criteria of evaluation for selected twenty (20) IoT platforms. Data collection process about the IoT platforms was supported by using ETE or Delphi technique. This model adopts the hybrid decision making approach and performs the quite accurate empirical analysis of IoT platforms by using CRITIC and TOPSIS. The proposed methodology prioritizes IoT platforms according to defined criteria. This model ranks Azure IoT platform at the top for SCM of COVID-19 vaccines. This platform can be installed by the SCM management team to perform their routine tasks related to vaccines management. Thus, the proposed model assists in selecting the most suitable and ideal option of IoT platform among the IoT offerings for the logistics and supply chaining of COVID-19 vaccines. This IoT platform can be leveraged to provide excursion notifications, vaccines monitoring, cold chain management, location awareness, routing tracking, shipping information etc. The first five model suggested by the proposed model are the right candidates for handling the Covid-19 vaccine. The results of proposed model are validated and verified in two different ways. The selected features in criteria are verified by using a survey with experts and the results are confirmed by SAW methods and expert groups. The evaluation metrics such as accuracy, precision and recall for the features validation are calculated. The values of precision, accuracy and recall 93%, 93% and 94% respectively. It indicates that the features selected in the evaluation criteria are covering the most essential aspects of IoT platforms. The overall performance of the proposed model is tested and checked based on five parameters such as security, usability, information and knowledge and effectiveness. This is survey based testing and results of this procedure suggests that the proposed assessment methodology can be used for evaluation and prioritization of IoT offerings that are intended for COVID-19 vaccine logistics and transportation. Hence, we conclude that this model is consistent and results are accurate and it can be applied to address the challenges faced in the logistics and transportation of COVID-19 vaccine. The model has the potential to perform well in practical scenario by tanking input of data about IoT platforms and it will evaluate platform based on the best possible features. It can save time and energy of managers by picking the most ideal IoT solution for their business needs. This model has the calibre to provide guideline for the IoT platform developers to update the functions and features of IoT platform with respect to vaccine management.

In future work, the authors are looking forward to apply the advanced and fuzzy decision making techniques for evaluating IoT platforms such as Fuzzy CRITIC, AHP and Fuzzy TOPSIS for designing a more sophisticated decision making model. Similarly, the evaluation parameters can also be updated to add more or new features of IoT platforms which are rapidly evolving due to the emerging situations in logistics and transportation of COVID-19 vaccine.

### Ethics declaration

This research study was approved by ethical committee of Shahzeb Shaheed Government Degree College, Razzar Swabi.

### Informed consent

All the expert groups/participants provided informed consent for the participation in this study.

## Data Availability

All data generated or analysed during this study are included in this published article. All the data is displayed in this manuscript.
